# The radiographic anatomy of the normal ovine digit, the metacarpophalangeal and metatarsophalangeal joints

**DOI:** 10.1007/s11259-012-9546-6

**Published:** 2012-11-23

**Authors:** Jennifer S. Duncan, Ellen R. Singer, Jane Devaney, Joanne W. H. Oultram, Anna J. Walby, Bridie R. Lester, Helen J. Williams

**Affiliations:** 1 School of Veterinary Science University of Liverpool, Leahurst Campus, Neston, Wirral, Cheshire CH64 7TE UK; 2 Institute of Ageing and Chronic Disease, University of Liverpool, Leahurst Campus, Neston, Wirral, Cheshire CH64 7TE UK

**Keywords:** Sheep, Lameness, Radiography, Anatomy

## Abstract

The aim of this project was to develop a detailed, accessible set of reference images of the normal radiographic anatomy of the ovine digit up to and including the metacarpo/metatatarsophalangeal joints. The lower front and hind limbs of 5 Lleyn ewes were radiographed using portable radiography equipment, a digital image processer and standard projections. Twenty images, illustrating the normal radiographic anatomy of the limb were selected, labelled and presented along with a detailed description and corresponding images of the bony skeleton. These images are aimed to be of assistance to veterinary surgeons, veterinary students and veterinary researchers by enabling understanding of the normal anatomy of the ovine lower limb, and allowing comparison with the abnormal.

## Introduction

Lameness in sheep flocks is a common and important source of poor welfare and economic loss worldwide. In England and Wales a recent study provided a prevalence estimate of lameness in sheep of 10.4 % (Kaler and Green 2008) and the cost of a single disease, namely footrot, is estimated at £24million annually (Nieuwhof and Bishop 2005). Lameness is painful (Ley et al. 1989, 1995), and dependent on the disease involved and the treatment strategy employed, it can be chronic and sustained in nature (FAWC 2011).

As a consequence of the economic value of commercial sheep, the diagnosis of lameness in sheep is often based only on veterinary or farmer clinical examination without the use of diagnostic imaging tools used in other species, such as radiography. In reality, physical examination is often adequate for determination of the diagnosis for many common causes of lameness (Hodgkinson 2010). However, as in other species, radiography can be a useful diagnostic and prognostic tool for certain causes of sheep lameness such as osteomyleitits, osteitis, deep digital sepsis, fractures and degenerative joint disease (Lovatt 2012; Scott and Sargison 2012). Due to developments in digital and computed radiography, radiography has become readily portable and less expensive diagnostic tool. Consequently, its use in sheep can now be justified economically in certain cases, particularly in sheep of higher economic or sentimental value, such as pedigree, research and pet sheep.

For accurate diagnosis from radiographic images, knowledge of the normal, radiographic anatomy of the structures under examination is required; however, for sheep, few, detailed, standard and published reference images of the normal radiographic anatomy of sheep are available for comparison with radiographs of sheep with suspected disease. Therefore, the aim of this study was to describe the normal radiographic anatomy of the digits and metacarpo/metatarsophalangeal joints of adult sheep.

## Materials and methods

This study was approved by the University of Liverpool Ethics Committee. All radiographic procedures were undertaken in accordance with the University of Liverpool Radiation Local Rules and Risk Assessment. All work involving animals was undertaken in accordance with EU Directive 2010/63/EU for animal experiments. (http://ec.europa.eu/environment/chemicals/lab_animals/legislation_en.htm)

The radiographic images were taken of the distal limbs (immediately below the carpus or tarsus) of one of a group of five Lleyn ewes, aged two years old. Each ewe was assessed to ensure they were not lame, and that their limbs were free from any external evidence injury or disease.

A portable direct radiography system (Superlight 80 generator, Veterinary X rays) with Kodak PQ Storage Phosphor screens 24 × 30 cm were used in the study. All radiographs were taken with the animal maintained in standing position, conscious and weight bearing on all four limbs. All images were taken with the long axis of the sheep limb perpendicular to the floor for consistency of positioning. Wooden blocks were used which were covered with sand paper to improve grip between the block and foot. The use of a long handled cassette holder ensured that the correct location and angle to the central beam could be maintained without motion, permitting images to be taken with minimal restraint of the sheep.

For each distal limb the following projections were used; lateromedial (LM), dorsopalmar (DP) and both dorsomedial-palmarolateral oblique (DMPLO) and dorsolateral-palmaromedial (DLPMO) oblique. Before any projections were taken, each leg and foot was brushed to remove potential artefacts. A consistent film focus distance 100 cm and an exposure of 62 kVp and 2.85 mAs were used throughout.

The radiographic images were imported into the image-processing software Visbion (Visbion Ltd, Version 4.0.21, Surrey UK). For each image the ewe identity, the image number, limb, view were recorded.

The images of the skeleton of the lower limbs were obtained from stripped and boiled out bones of a non lame cull ewe. The three dimensional computerised tomography (CT) images were acquired using a GE Lightspeed RT16 CT scanner (GE Medical Systems Ltd, Hertfordshire UK), exposures of 150 kV and 250 mAs, and Osirix Imaging Software (http://www.osirix-viewer.com/index.html).

## Results

**Fig. 1:**
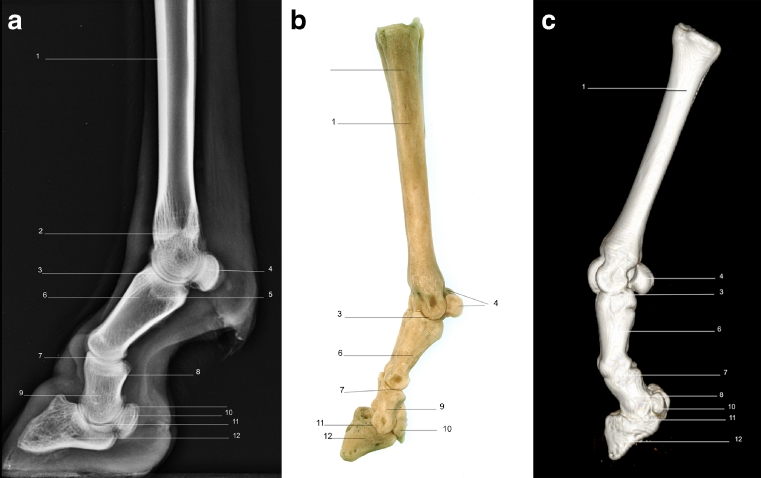
**a** Radiograph of lateromedial view of left hind limb. **b** Bone skeleton of left hind limb lateral view **c** CT 3D image of left fore limb lateral view. *1*. Fused metacarpal bones 3 and 4. *2*. Scar of epiphysis of metacarpus. *3*. Metacarpophalangeal joint. *4*. Proximal sesamoid bones. *5*. Scar of epiphysis of proximal phalanx. *6*. Proximal phalanx. *7*. Proximal interphalangeal joint. *8*. Scar of epiphysis of middle phalanx. *9*. Middle phalanx. *10*. Distal sesamoid bone. *11*. Distal interphalangeal joint. *12*. Distal phalanx

**Fig. 2:**
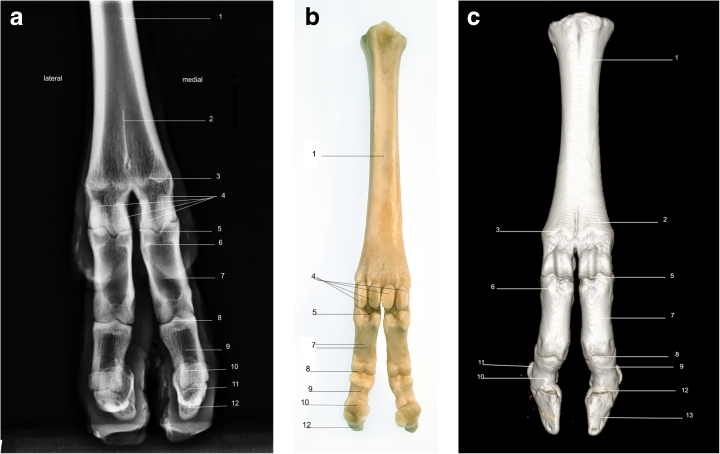
**a** Radiograph of dorsoplantar view of left hind limb. **b** Bone skeleton of left hind limb palmar view **c** CT 3D image of left forelimb dorsal view. *1*. Fused metacarpal bones 3 and 4. *2*. Sagittal septum. *3*. Scar of epiphysis of metacarpus. *4*. Proximal sesamoid bones. *5*. Metacarpophalangeal joint. *6*. Scar of epiphysis of proximal phalanx. *7*. Proximal phalanx. *8*. Proximal interphalangeal joint. *9*. Middle phalanx. *10*. Distal sesamoid bones. *11*. Distal interphalangeal joint. *12*. Distal phalanx

**Fig. 3:**
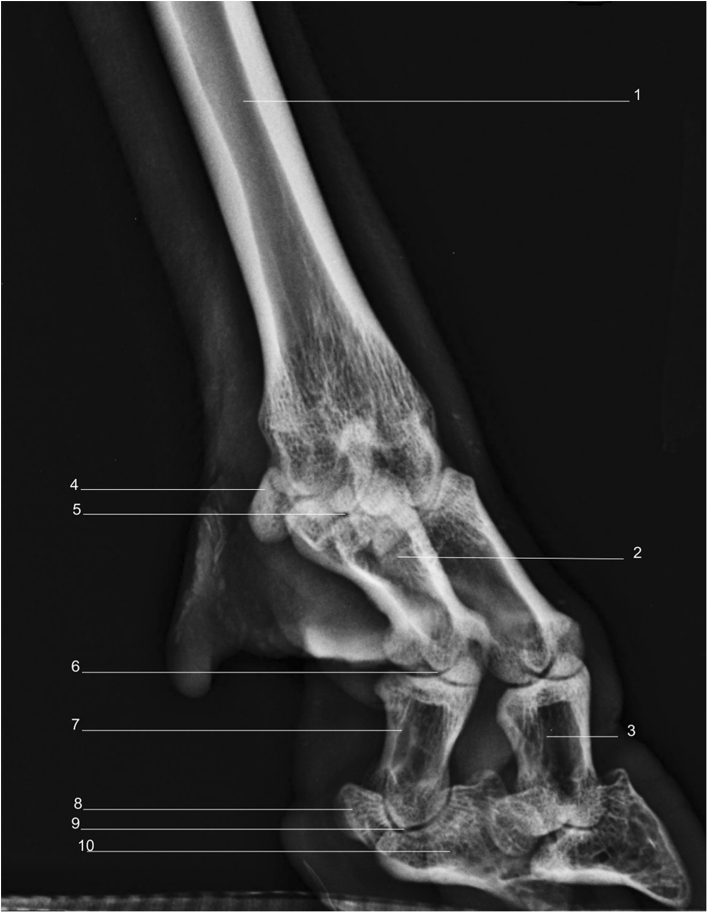
Radiograph of dorsolateral –palmaromedial oblique view of right forelimb. *1*. Fused metacarpal bones 3 and 4. *2*. Proximal phalanx. *3*. Middle phalanx. *4*. Lateral proximal sesamoid. *5*. Metacarpophalangeal joint. *6*. Lateral proximal interphalangeal joint. *7*. Lateral middle phalanx. *8*. Lateral distal sesamoid bone. *9*. Lateral distal interphalangeal joint. *10*. Lateral distal phalanx

**Fig. 4:**
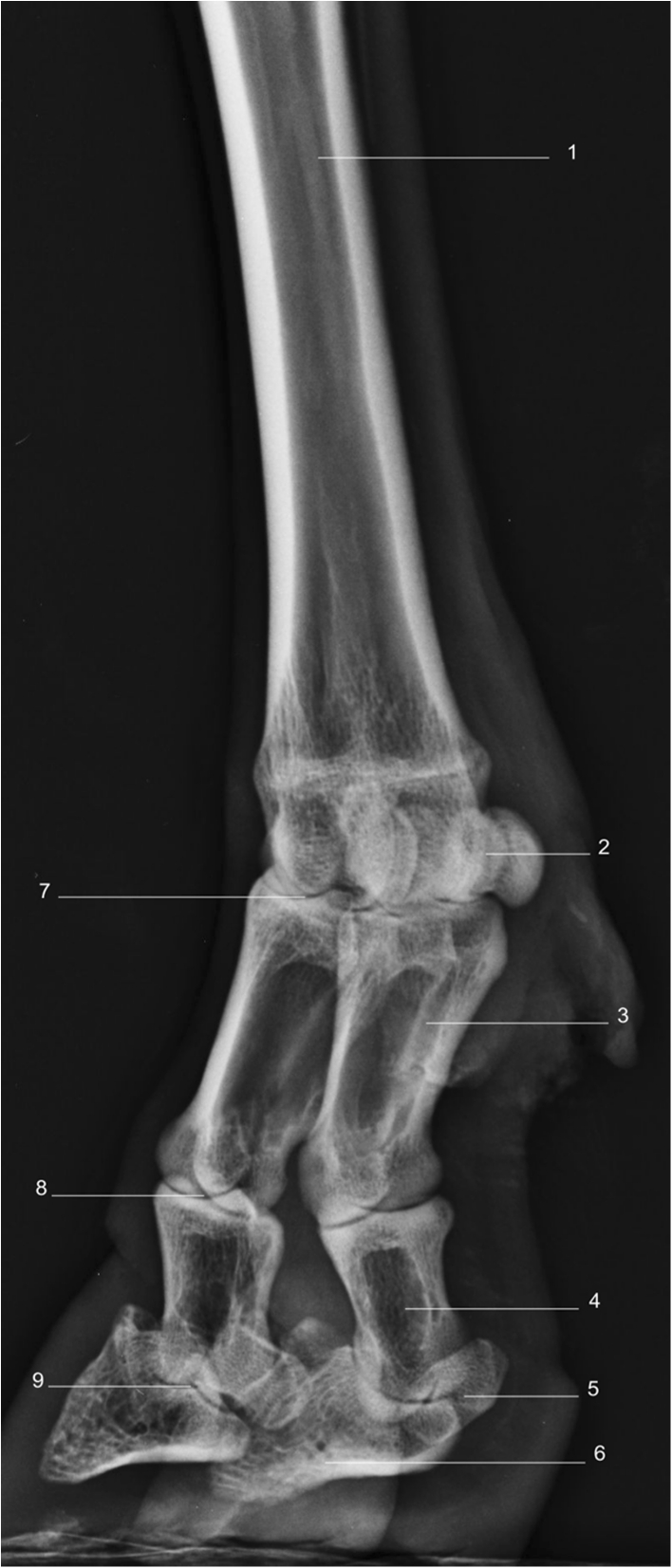
Radiograph of dorsomedial — palmarolateral oblique view of right forelimb. *1*. Metacarpus. *2*. Medial Proximal sesamoids. *3*. Medial Proximal phalanx. *4*. Medial Middle phalanx. *5*. Medial Distal sesamoid bone. *6*. Medial Distal phalanx. *7*. Lateral Metacarpal phalangeal joint. *8*. Lateral Proximal interphalangeal joint. *9*. Lateral Distal interphalangeal joint

Using a radiographic plate 24 cm × 30 cm, it was feasible to acquire diagnostic quality images from the midcarpus/tarsus down to and including the foot, using the standard projections of LM (Fig. 1a and b), DP (Fig. 2a and b), DMPLO (Fig. 3) and DLPMO (Fig. 4) in conscious sheep.

Radiographic images were obtained for the front and hind limbs distal to the metacarpal/metatarsal phalangeal joints (Fig. 1a, 2a, 3 and 4). Each image was described in terms of its anatomical features and for illustrative purposes is accompanied by a photograph of the gross anatomy of the corresponding skeletal structures (Figs. 1b and 2b). A CT image of the LM (Fig. 1c) and DP (Fig. 2c) views of the front limb is also provided.

### Distal phalanx

**Fig. 5:**
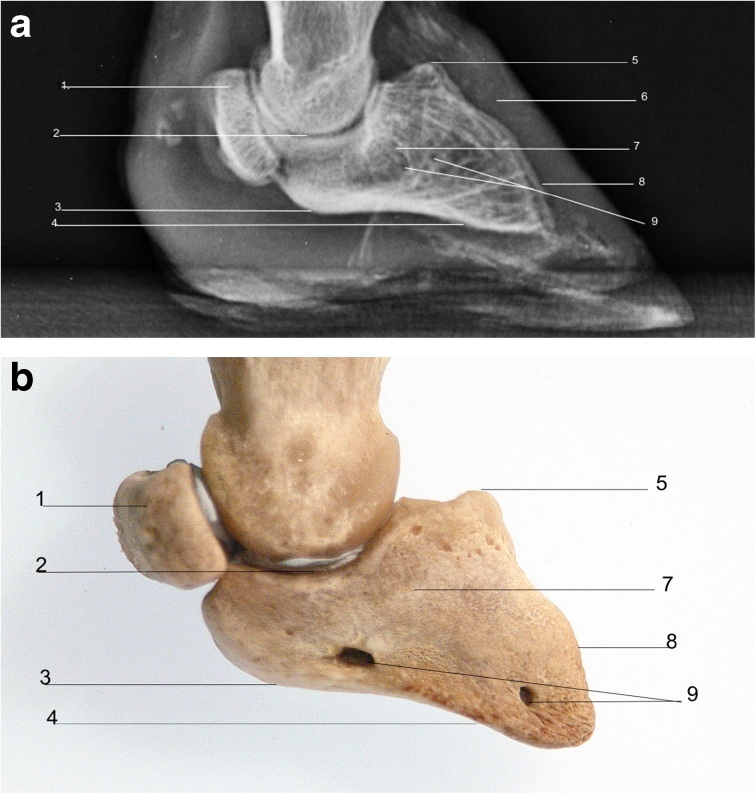
**a** Radiograph of right forelimb hoof, lateromedial view. **b** Bones of the right forelimb hoof lateral view. *1*. Distal sesamoid bone. *2*. Distal interphalangeal joint. *3*. Tubercle of attachment of deep digital flexor tendon. *4*. Solar surface. *5*. Extensor process. *6*. Hoof capsule. *7*. Distal phalanx. *8*. Dorsal surface of distal phalanx. *9*. Nutrient foramen

The shape of distal phalanx reflects the shape of the hoof (Fig. 5a and b). It has articular, dorsal and solar surfaces. The extensor process is the attachment site for the common digital extensor tendons and dorsal ligament of the proximal interphalangeal joint. The deep digital flexor tendon inserts on a tubercle on the palmar/plantar border of the distal phalanx. The nutrient foramen of the common digital artery can be seen as a circular radiolucent area axially to the extensor process. Proximally, the distal phalanx articulates with the middle phalanx and the articular surface bears a sagittal ridge which meets with a sagittal groove on the articular surface of the middle phalanx.

### Distal sesamoid bones

The medial and lateral distal sesamoid bones sit in the palmar/plantar aspect of their respective distal interphalangeal joints and articulate with both the middle (proximodorsally) and distal phalanges (dorsodistally) (Figs. 10a and b, 11a and b). In a DP view the distal sesamoid bone is superimposed on to the middle phalanx at the level of distal interphalangeal joint. From this view, the sesamoid bones are approximately rectangular in shape. On the LM view the distal sesamoid bones of the lateral and medial digits are superimposed. The distal sesamoid bones are attached by ligaments to both the proximal and middle phalanges, which are not apparent radiographically. The deep digital flexor tendons pass over the distal sesamoid bones.

### Proximal and middle phalanges

**Fig. 6:**
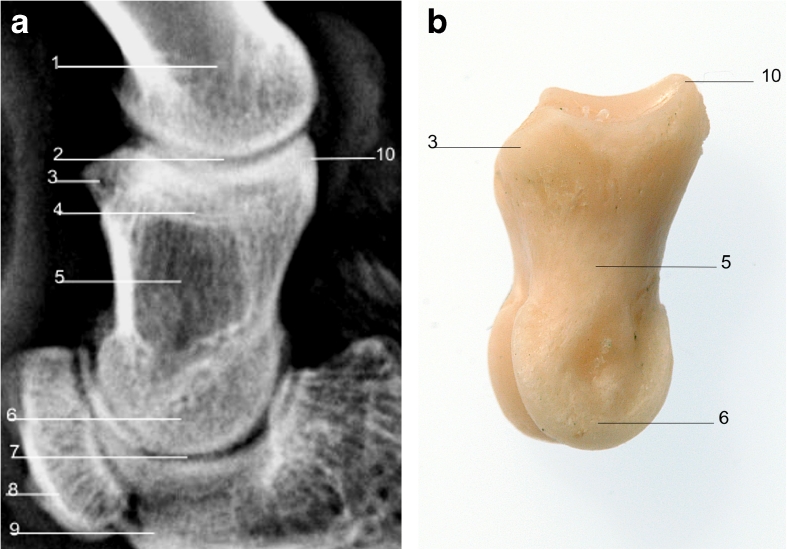
**a** Radiograph of lateromedial view of right forelimb showing radiographic anatomy of the middle phalanx. **b** Middle phalanx lateral view. *1*. Proximal phalanx. *2*. Proximal interphalangeal joint. *3*. Tuberosity for attachment of inter-phalangeal ligament. *4*. Scar of epiphysis of middle phalanx. *5*. Middle phalanx. *6*. Articular condyle. *7*. Distal interphalangeal joint. *8*. Distal sesamoid bone. *9*. Distal phalanx. *10*. Extensor process for attachment of common digital extensor tendon

**Fig. 7:**
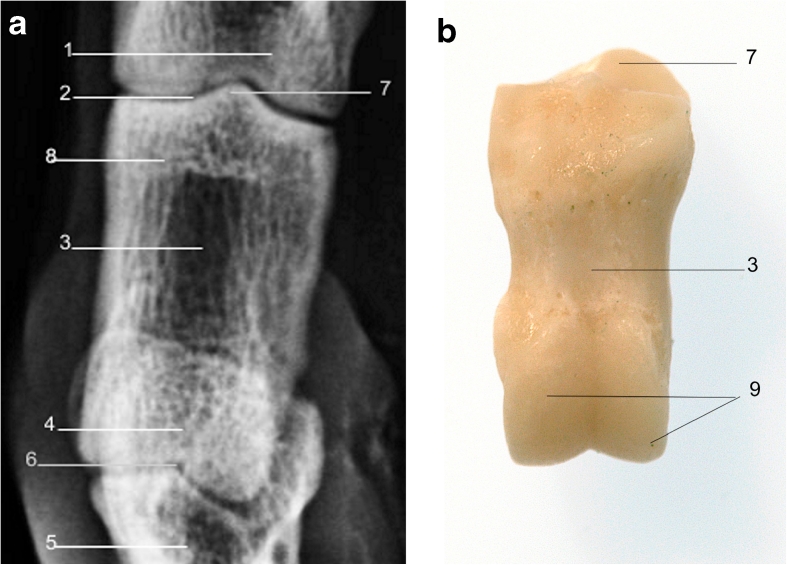
**a** Radiograph of dorsopalmar view of right forelimb showing radiographic anatomy of the middle phalanx. **b** Middle phalanx palmar view. *1*. Proximal phalanx. *2*. Proximal interphalangeal joint. *3*. Middle phalanx. *4*. Distal sesamoid bone. *5*. Distal phalanx. *6*. Distal interphalangeal joint. *7*. Sagittal ridge of middle phalanx. *8*. Scar of epiphysis of middle phalanx. *9*. Articular condyles of middle phalanx

**Fig. 8:**
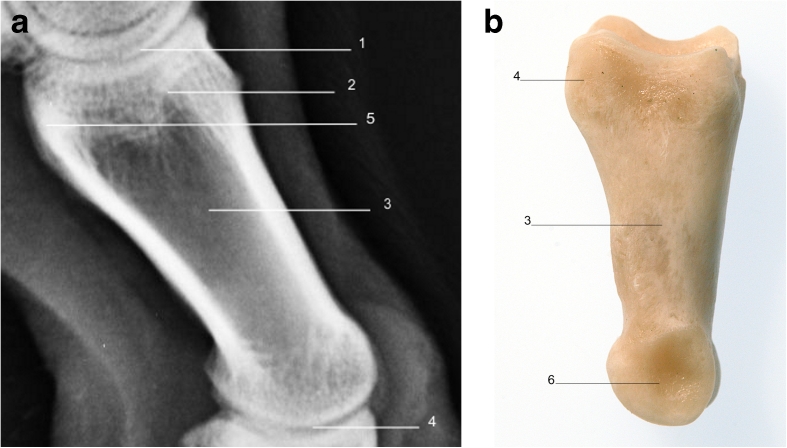
**a** Radiograph of lateral view of right forelimb showing radiographic anatomy of the proximal phalanx. **b** Proximal phalanx lateral view. *1*. Metacarpophalangeal joint. *2*. Scar of epiphysis of proximal phalanx. *3*. Proximal phalanx. *4*. Proximopalmar tubercle. *5*. Proximal interphalangeal joint. *6*. Articular condyle

**Fig. 9:**
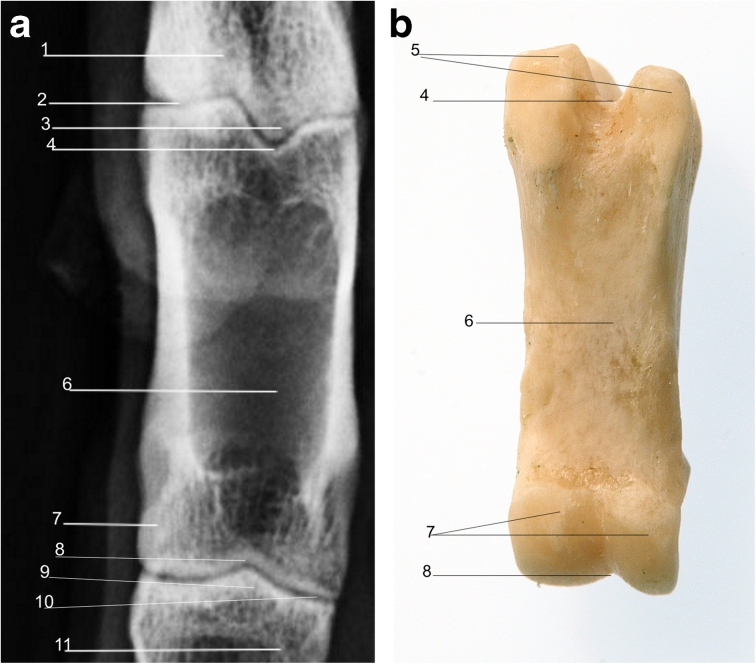
**a** Radiograph of dorsopalmar view of right fore limb showing radiographic anatomy of the proximal phalanx. **b** Proximal phalanx palmar view. *1*. Metacarpus. *2*. Metacarpophalangeal joint. *3*. Sagittal ridge. *4*. Sagittal groove. *5*. Proximopalmar tubercle. *6*. Proximal phalanx. *7*. Articular condyle. *8*. Sagittal groove. *9*. Sagittal ridge. *10*. Proximal interphalangeal joint. *11*. Middle phalanx

The proximal and middle phalanges are similar in their rectangular shape in LM and DP views; however, the proximal phalanx is generally twice the length of the distal phalanx (Figs. 6a, 7, 8, and 9b).

In young animals, both bones have a proximal epiphysis which may be visible radiographically in the skeletally immature animal, or the scar of which may be visible in when the epiphysis has fused on maturity. The proximal phalanx has axial and abaxial proximopalmar tubercles for attachment of proximal sesamoid ligaments (Fig. 8a and b), and the middle phalanx has single abaxial proximopalmar tubercles for attachment of the interphalangeal ligament (Fig. 6a and b). The distal articular surfaces of both the proximal and middle phalanges have a sagittal groove which fits with the corresponding sagittal ridge of the proximal articular surface of the corresponding middle phalanx or distal phalanx with which they articulate.

### Proximal sesamoid bones

**Fig. 10:**
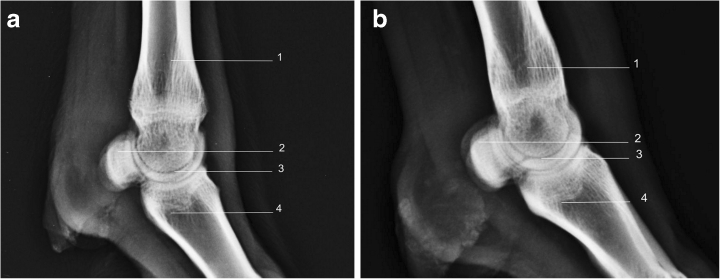
**a** Radiograph of metacarpal phalangeal joint, lateral view right fore limb. *1*. Metacarpus. *2*. Proximal sesamoid bones. *3*. Metacarpal phalangeal joint. *4*. Proximal phalanx. **b** Radiograph of metatarsophalangeal joint, lateral view right hind limb. *1*. Metatarsus. *2*. Proximal sesamoid bones. *3*. Metatarsal phalangeal joint. *4*. Proximal phalanx

**Fig. 11:**
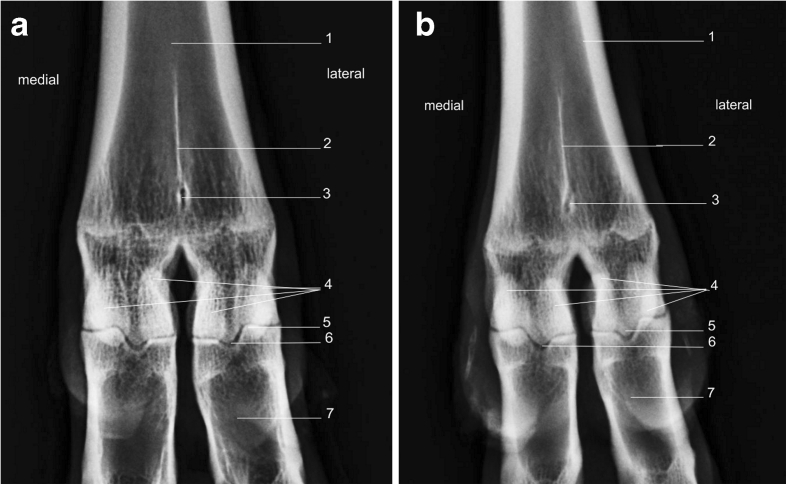
**a** Radiograph of metacarpal phalangeal joint, dorsopalmar view right fore limb. *1*. Metacarpus. *2*. Sagittal septum. *3*. Nutrient foramen. *4*. Proximal sesamoid bones. *5*. Metacarpal phalangeal joint. *6*. Midsagittal ridge. *7*. Proximal phalanx. **b** Radiograph of metatarsal phalangeal joint, lateral view right hind limb. *1*. Metatarsus. *2*. Line of fusion. *3*. Nutrient foramen. *4*. Proximal sesamoid bones. *5*. Metatarsal phalangeal joint. *6*. Midsagittal ridge. *7*. Proximal phalanx

There are two pairs of proximal sesamoid bones, one pair on each distal metacarpus/metatarsus phalangeal joints (Fig. 10a, b, 11a and b). They articulate with the palmar medial and lateral condyles of the third and fourth metacarpal (metatarsal) bones, each other and the base of each proximal phalanx. The sesamoid bones are attached to the other bones via collateral and distal sesamoidean ligament. In a DP view the proximal sesamoid bones appear oval in shape; however, on a LM view their appearance is D-shaped (Figs. 10a and 11a.)

### Radiographic anatomy of the metacarpophalangeal joint and metatarsophalangeal joint

The metacarpophalangeal joint and the metatarsophalangeal joints are commonly known as the fetlock joints. They form the junction between the distal ends of the third and fourth metacarpal (tarsal) bones, the articular surfaces of the lateral and medial proximal phalanges and the proximal sesamoid bones (Fig. 10a, b, 11a and b). The joints of the hind and forelimbs are anatomically indistinct. They are simple hinge joints, capable of only flexion and extension, and the opposing proximal phalangeal sagittal grooves and distal metacarpal sagittal ridges on the articular surfaces of the bones limit medial and lateral movement.

The proximal interphalangeal joint (Figs. 6a and 7a), known as the pastern joint, occurs between the proximal and middle phalanx. It is a simple saddle joint, allowing the movements of extension and flexion, again the sagittal groove and ridge system helps to limit medial and lateral movement. The distal interphalangeal joint, sits entirely within the hoof capsule (Fig. 5a and b) and is often referred to as the coffin joint. It is also a simple saddle joint connecting the middle and distal phalanges with the distal sesamoid bone which sits proximally.

## Discussion

Using portable radiographic equipment and digital image processing it is possible to obtain diagnostic quality radiographic images of a sheep’s lower limb at a relatively low cost in a standing unsedated sheep. This portable and economic aid is likely to be of use as a diagnostic and prognostic tool in higher value animals, such as pedigree and breeding stock, sheep kept as pets or those used in research for diagnosis of disease involving internal hoof structures and skeletal pathology.

The output of this study, a radiographic library of images and descriptive analysis of images, should be of benefit to veterinary surgeons, veterinary students and researchers by enabling comparison of radiographs from lame and non lame sheep.
